# nAChRs Mediate Human Embryonic Stem Cell-Derived Endothelial Cells: Proliferation, Apoptosis, and Angiogenesis

**DOI:** 10.1371/journal.pone.0007040

**Published:** 2009-09-15

**Authors:** Jin Yu, Ngan F. Huang, Kitchener D. Wilson, Jeffrey B. Velotta, Mei Huang, Zongjin Li, Andrew Lee, Robert C. Robbins, John P. Cooke, Joseph C. Wu

**Affiliations:** 1 Department of Cardiovascular Medicine, Xijing Hospital, Fourth Military Medical University, Xi'an, People's Republic of China; 2 Department of Medicine, Division of Cardiovascular Medicine, Stanford University School of Medicine, Stanford, California, United States of America; 3 Department of Radiology and Molecular Imaging Program at Stanford (MIPS), Stanford University, Stanford, California, United States of America; 4 Department of Cardiothoracic Surgery, Stanford University School of Medicine, Stanford, California, United States of America; Universidade Federal do Rio de Janeiro (UFRJ), Instituto de Biofísica da UFRJ, Brazil

## Abstract

**Background:**

Many patients with ischemic heart disease have cardiovascular risk factors such as cigarette smoking. We tested the effect of nicotine (a key component of cigarette smoking) on the therapeutic effects of human embryonic stem cell-derived endothelial cells (hESC-ECs).

**Methods and Results:**

To induce endothelial cell differentiation, undifferentiated hESCs (H9 line) underwent 4-day floating EB formation and 8-day outgrowth differentiation in EGM-2 media. After 12 days, CD31^+^ cells (13.7±2.5%) were sorted by FACScan and maintained in EGM-2 media for further differentiation. After isolation, these hESC-ECs expressed endothelial specific markers such as vWF (96.3±1.4%), CD31 (97.2±2.5%), and VE-cadherin (93.7±2.8%), form vascular-like channels, and incorporated DiI-labeled acetylated low-density lipoprotein (DiI-Ac-LDL). Afterward, 5×10^6^ hESC-ECs treated for 24 hours with nicotine (10^−8^ M) or PBS (as control) were injected into the hearts of mice undergoing LAD ligation followed by administration for two weeks of vehicle or nicotine (100 µg/ml) in the drinking water. Surprisingly, bioluminescence imaging (BLI) showed significant improvement in the survival of transplanted hESC-ECs in the nicotine treated group at 6 weeks. Postmortem analysis confirmed increased presence of small capillaries in the infarcted zones. Finally, *in vitro* mechanistic analysis suggests activation of the MAPK and Akt pathways following activation of nicotinic acetylcholine receptors (nAChRs).

**Conclusions:**

This study shows for the *first* time that short-term systemic administrations of low dose nicotine can *improve* the survival of transplanted hESC-ECs, and enhance their angiogenic effects *in vivo*. Furthermore, activation of nAChRs has anti-apoptotic, angiogenic, and proliferative effects through MAPK and Akt signaling pathways.

## Introduction

Since the first description of the isolation and expansion of human embryonic stem cells (hESCs) in 1998[1], [2], there has been tremendous excitement over the potential clinical and therapeutic applications of hESC derivatives[3], [4]. Derivatives of hESCs offer potential effective treatments for intractable diseases such as heart failure, neurological injury, and diabetes. Accordingly, numerous protocols have been developed for the differentiation of endothelial cells[5], [6], [7], [8], [9] [14]–[16], cardiomyocytes[10], neurons[11], and pancreatic islet cells[12], among other cell types.

In particular, hESC-derived endothelial cells (hESC-ECs) are a promising cell source for the treatment of a variety of ischemic diseases, including stroke, myocardial ischemia, and peripheral vascular disease[6], [13], [14]. However, many significant hurdles must be overcome before hESC-EC regeneration can become clinically relevant, including massive cell death and apoptosis following transplantation[15], teratoma formation[16], and immune rejection[17] by the host organism.

Cardiovascular (CV) risk factors, including cigarette smoke, are known to reduce the number and function of adult endothelial progenitor cells (EPCs) in humans[18]. We are interested in understanding how one such known major CV risk factor, cigarette smoke, would affect the function of hESC-ECs. Thus, this study focused on the effects of nicotine, the primary addictive component among 4000 known constituents of tobacco smoke, on hESC-ECs.

## Materials and Methods

### Preparation of hESC-Ecs

Karyotypically normal hESCs (H9 line from Wicell, passages 35–45) were maintained in the undifferentiated state on an inactivated mouse embryonic fibroblast (MEF) feeder layer as previously described[19]. The medium consisted of Dulbecco's modified Eagle's medium (DMEM)/F-12 (Invitrogen, CA), 20% knockout serum replacement (Invitrogen, CA), 0.1 mM nonessential amino acids (Invitrogen, CA), 2 mM L-glutamine (Invitrogen, CA), 0.1 mM β-mercaptoethanol (Sigma, MO), and 8 ng/ml basic fibroblast growth factor (bFGF, Invitrogen, CA). Prior to endothelial differentiation, hESCs were seeded onto Growth Factor Reduced (GFR) Matrigel-coated plates (BD Biosciences, CA) in mTeSR medium (StemCell Technologies Inc., Vancouver, Canada) for MEF deprivation. To initiate hESC differentiation into human embryoid bodies (EBs), the undifferentiated hESCs were transferred to ultra-low attachment plates (Corning Incorporated, Corning, NY) for 4 days and cultured in differentiation medium containing Iscove's modified Dulbecco's medium (IMDM) (Invitrogen, CA), 20% defined fetal bovine serum (FBS) (Hyclone, UT), 0.1 mM nonessential amino acids, 2 mM L-glutamine, 450 µM monothioglycerol (Sigma), 50 U/ml penicillin (Sigma), and 50 µg/ml streptomycin (Sigma). After 4 days, hEBs were transferred to a 0.1% gelatin coated dish and grown for an additional 8 days in endothelial cell growth medium-2 (EGM-2, Clonetics Corp., CA) that contains 2% FBS, 0.04% hydrocortisone, 0.1% heparin, 0.1% human epidermal growth factor (hEGF), 0.1% long R3-human insulin-like growth factor (IGF-1), 0.1% ascorbic acid, 0.4% human fibroblast growth factor (hFGF)-B, 0.1% vascular endothelial growth factor (VEGF), 0.05% gentamicin, and 0.05% amphotericin-B.

### Flow cytometry cell sorting of hESC-Ecs

After 12 days of differentiation, flow cytometry sorting was used to purify hESC-ECs. Single cell suspensions were obtained by treating cell cultures with PBS-based cell dissociation buffer (Invitrogen, CA) at 37°C for 30 min. Cells were then passed through a 40-µm cell strainer (BD Falcon, San Diego) and incubated for 30 min at 4°C with mouse phycoerythrin (PE)-conjugated anti-human CD31 (BD Bioseciences). CD31^+^ cells were isolated using FACScan (Becton Dickinson). Selection for PE-conjugated CD31^+^ endothelial cells was carried out by first gating on larger cells within a SSC-A and FSC-A dot plot to exclude non-viable cells and debris. The CD31^+^ cell population was then identified by PE^+^ expression compared to unstained control cells. To expand the isolated hESC-ECs, CD31^+^ cells were grown on 0.1% gelatin-coated plates in EGM-2, changing the medium every 2 days. After passage 3, we again performed flow cytometry sorting with CD31 antibody to further purify hESC-ECs for subsequent cultures. In order to study the phenotype of CD31^+^ cells thus obtained, we confirmed the endothelial phenotype of our CD31^+^ twice-purified hESC-ECs by flow cytometry analysis for the expression of vWF, CD31, and VE-cadherin (BD Biosciences), DiI-ac-LDL uptake assay, and Matrigel tube formation assay as previously described [15].

### pUb-Fluc-eGFP transduction of hESCs

To track transplanted cells *in vivo*, we used an H9 hESC line that stably expresses a double fusion (DF) reporter gene containing firefly luciferase (Fluc) and enhanced green fluorescence protein (eGFP)[15]. After fixing with 4% paraformaldehyde in PBS for 15 minutes and incubating with 4% normal goat serum for 30 minutes to block nonspecific binding, immunostaining was performed with Oct-4 antibody (Santa Cruz Inc., CA) on both non-transduced control hESCs and ^DF+^hESCs to test the effect of reporter gene expression on hESC phenotype. Cells were then incubated with Alexa 594-conjugated rabbit anti-goat secondary antibodies (Invitrogen, CA) for 30 minutes and nuclear counterstained with 4, 6-diamidino-2- phenylindole (DAPI). Images were obtained with a Zeiss Axiovert microscope (Sutter Instrument Co., USA). To test the effect of transduced reporter gene on cellular proliferation, as well as the effect of nAChRs activation on reporter gene expression, control (non-transduced) hESC-ECs and stably transduced ^DF+^hESC-ECs were plated uniformly in 96-well plates at a density of 5,000 cells per well, in the presence of different nicotine concentrations (10^−8^, 10^−6^, 10^−4^, or 10^−2^ M) at 24, 48, and 72 hour time points, respectively. At each indicated time point, MTS/PMS solution (Promega) was added to each well for 3 hours. The optical density (O.D.) of each well was measured on an ELISA micro-plate reader at 490 nm, according to a previously published protocol[20]. Ten samples per group were assayed and averaged. Experiments were performed in triplicate.

### Cardiac transplantation of ^DF+^hESC-Ecs

Eight-week-old female SCID Beige mice (23–27 g, Charles River Laboratories, Inc.) were stratified to one of four groups that underwent aseptic lateral thoracotomy and ligation of the left anterior descending (LAD) coronary artery as described[21]. Group 1 animals (n = 5) received intramyocardial injections of 5×10^6 DF+^hESC-ECs in 30 ul of 10^−8^ M nicotine solution; cells had been pretreated overnight with 10^−8^ M nicotine. Postoperatively, animals received daily administrations of nicotine in the drinking water (which was a 2% saccharine solution, with nicotine in a concentration of 100 ug/ml, administered per libitum)[22]. Group 2 animals (n = 5) received intramyocardial injections of non-pretreated ^DF+^hESC-ECs suspended in 30 ul PBS, as well as 2% saccharine in their drinking water post-surgery but no nicotine treatment. Group 3 animals (n = 5) did not receive cell injections but rather intramyocardial injections with 30 ul of 10^−8^ M nicotine solution, as well as oral administrations of nicotine in the drinking water as described above (negative control for group 1). Group 4 animals (n = 5) received intramyocardial injections with 30 ul of phosphate buffered saline (PBS) and 2% saccharine in drinking water after surgery (negative control for group 2). Harvested ^DF+^hESC-ECs were kept on ice for <30 min for optimal viability. Serum nicotine levels were measured as previously described[22]. Animals that recovered uneventfully underwent bioluminescence imaging (BLI) later. Study protocols were approved by the Stanford Animal Research Committee.

### Optical BLI of transplanted ^DF+^hESC-Ecs

At specific timepoints, BLI was performed using the Xenogen In Vivo Imaging System (IVIS 200, Xenogen, Alameda, CA). Animals received isoflurane (2%) for general anesthesia. After intraperitoneal injection of the reporter probe D-luciferin (375 mg/kg body weight), animals were imaged for 30 minutes with 1-minute acquisition intervals. The same mice were scanned for 6 weeks. BLI images were analyzed using the Igor image analysis software (Wavemetrics, Lake Oswego, OR). Regions of interest (ROIs) were drawn over the signals, and BLI was quantified in units of maximum photons per second per centimeter square per steradian (p/s/cm^2^/sr) as described [16].

### Postmortem histology

Animals were sacrificed for postmortem histology study at week 6. The hearts were embedded in Optical Cutting Temperature (OCT) compound (Tissue-Tek Sakura Finetek, CA) and snap frozen in liquid nitrogen. Five-micron sections were cut in both the proximal and apical regions of the infarct zone. To trace the transplanted ^DF^hESC-ECs in the ischemic heart, slides were double stained for GFP (Molecular Probes) and human-specific CD31 (BD Pharmingen). Sections were counterstained with DAPI. Cell engraftment was confirmed by identification of GFP and human-specific CD31 expression under fluorescent microscopy. To detect mouse microvascular density (MVD) in the peri-infarct area, immunohistochemical staining for mouse-specific CD31 was carried out by using the Biocare Medical Universal HRP-DAB kit (Biocare Medical, Walnut Creek, CA) as described[23]. The staining was performed and number of capillary vessels was counted in 10 randomly selected areas using a light microscope (x200 magnification).

### Capillary-like tube formation and cell apoptosis study *in vitro*

In order to further study the dose-dependent effect of nicotine on hESC-EC angiogenesis under hypoxic conditions, 24-well plates were coated with growth factor reduced (GFR) Matrigel and equilibrated with growth factor and serum-deprived EBM-2 medium[24]. Cells (1×10^5^) were seeded into each well containing different concentrations of nicotine (10^−8^, 10^−6^, 10^−4^, or 10^−2^ M) followed by incubation in conditions of normoxia (5% CO_2_, 21% O_2_, and 74% N_2_) or hypoxia (5% CO_2_, 1% O_2_, and 94% N_2_). Development of tube formation was assessed after 48 hours using an inverted phase-contrast microscope (Zeiss AxioVert 100 M; Carl Zeiss Inc.). Images were captured with a video graphic system (Zeiss AxioCam; Carl Zeiss Inc.). Relative tube-like formation was determined by measuring the length of tube-like structures in five random fields from each well and expressed as percent of vehicle-treated cells [25]. To determine whether nicotine can improve the survival of hESC-ECs by reducing cellular apoptosis during hypoxia, hESC-ECs were cultured for 24 and 48 hours in growth factor and serum-deprived EBM-2 medium, at 37°C under hypoxic conditions (5% CO_2_, 1% O_2_, and 94% N_2_). The proportion of apoptotic hESC-ECs in comparison to the total hESC-ECs was determined by staining with PE-conjugated Annexin-V (Sigma, MO), and the number of dead cells was determined by staining with 7-AAD, as previously described [26]. Stained cells were analyzed by flow cytometry (BD LSR cell analyzer, San Jose, CA).

### Immunoblotting

hESC-ECs were incubated with or without nicotine in normoxia (5% CO_2_, 21% O_2_, and 74% N_2_) or hypoxia (5% CO_2_, 1% O_2_, and 94% N_2_) for 48 h. Samples (n = 3) were then rinsed in chilled PBS before lysing with RIPA buffer (Pierce) containing protease inhibitors. Proteins were quantified by BCA protein assay kit (Pierce) for equal loading. Sodium dodecyl sulfate polyacrylamide gel electrophoresis (SDS-PAGE) was carried out using NuPage 4–12% Bis-Tris pre-cast gels (Invitrogen), and the samples were then transferred onto nitrocellulose membranes. The membranes were blocked in 5% BSA before incubating with the following monoclonal antibodies: HIF-1α (Novus Biologicals), pAkt1 (Cell Signaling Technology), Akt1 (Cell Signaling Technology), pMAPK (Cell Signaling Technology), MAPK (Cell Signaling Technology), or total actin (Sigma). Horse radish peroxidase (HRP)-conjugated anti-rabbit (GE) secondary antibody was applied, and the proteins were visualized by an ECL Detection Kit (Amersham). Protein quantities for pMAPK and pAkt1 were normalized to total MAPK and Akt1, respectively. HIF quantities were normalized by total actin abundance. Data was quantified using Image J software (NIH, Bethesda, MD).

### RNA isolation, reverse transcription, and quantitative polymerase chain reaction

Total RNA isolation was carried out with the RNEasy Kit (Qiagen) with modifications. Samples were lysed in Trizol (Invitrogen) before adding 200 µl chloroform per ml Trizol to each sample and then centrifuging for phase separation. The aqueous phase containing RNA was removed and combined with 3.5x the volume of RLT lysis buffer and 2.5x ethanol. The mixture was then applied to an RNeasy mini column, and purified RNA was obtained following the manufacturer's instructions. First strand DNA was synthesized by Superscript II reverse transcriptase (Invitrogen) according to the manufacturer's instruction. Taqman real-time PCR assays for VEGF-A, bFGF, and nAChRs α1, α5, α7, and α9 were purchased from Applied Biosystems. Oligonucleotides for 18S were generated based on sequences from Shetzline *et al.* [27]. Real-time PCR reactions were performed on a 7300 Real-Time PCR system (Applied Biosystems) for 40 cycles. The data were assessed by the ΔΔCt method[28], normalized to 18S housekeeping gene, and expressed as relative fold changes.

### Bromodeoxyuridine (BrdU) Incorporation

To examine the effect of nicotine on cell proliferation in 1% O_2_ conditions, hESC-ECs were cultured in the presence of EBM containing 10^−8^ M nicotine for up to 2 days. At the indicated time points, the samples were pulsed by BrdU for 2 hours and then fixed in ethanol (n = 3). The samples were then immunofluorescently stained for BrdU expression using a fluorescein-conjugated detection kit according to the manufacturer's instructions (Roche Applied Sciences, Indianapolis, IN). Hoechst 33342 nuclear dye was then applied to visualize total nuclei. Images were acquired with 20X objectives, and the percentage of BrdU-expressing cells was quantified out of at least 300 cells using Image J software.

### Akt Inhibition Assay

To verify the role of Akt in modulating nicotine's enhacement in cell survival under hypoxic conditions, hESC-ECs were cultured in EBM in 1% O_2_ hypoxia in the presence or absence of 10^−8^ M nicotine. After 24 h, the cells were treated with 5 µM Akt IV inhibitor (Calbiochem), 10^−8^ M Nicotine, or 5 µM Akt IV inhibitor +10^−8^ M Nicotine for 1 h in hypoxia (n = 4). Cells were assessed for viability using the Live/Dead cytotoxicity assay (Invitrogen) in which live cells could incorporate calcein-AM (green), whereas dead cells were labeled by ethidium homodimer (red). Samples were then imaged by fluorescence microscopy with 10X objectives.

### Statistical analysis

All results are expressed as mean ± standard deviation, except where defined elsewise. Statistical significance was tested was performed by the Student's t-test for comparison of 2 groups or one-way analysis of variance (ANOVA) with Holm's adjustment for multiple comparisons. Statistical significance was accepted at *P*<0.05.

## Results

### Differentiation and characterization of hESC-Ecs

**Figure 1:**
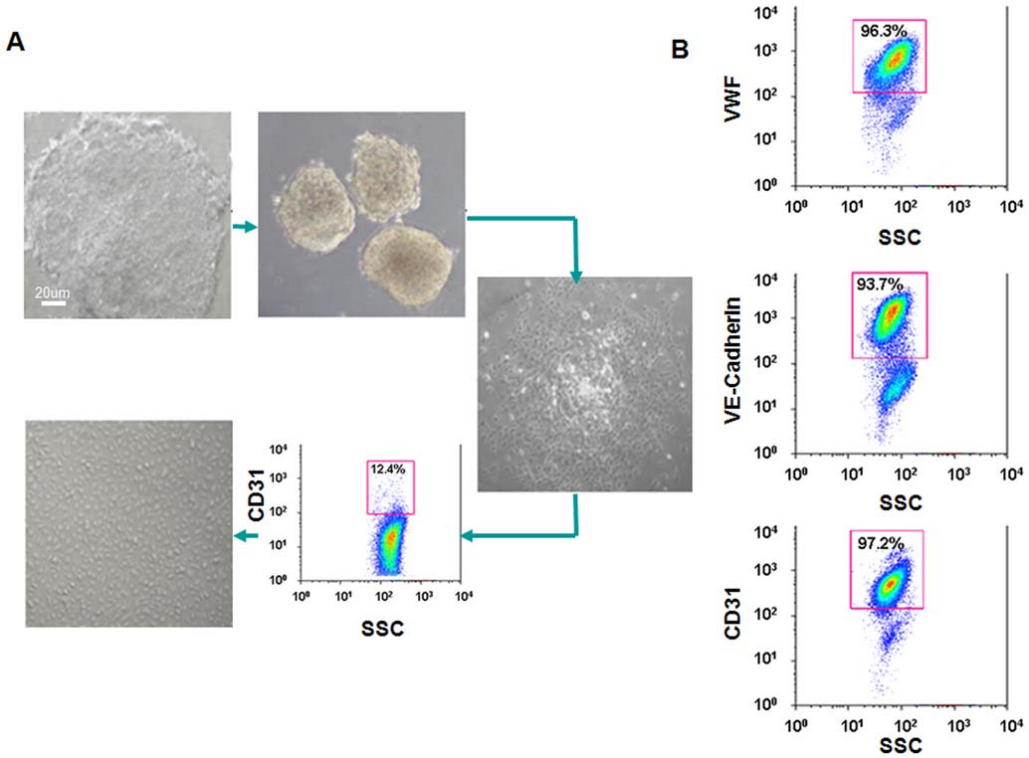
Differentiation of hESCs to an endothelial lineage. (A) hESCs were allowed to form embryoid bodies (EB) for 4 days in differentiation medium and ultra-low attachment dishes. EBs were then transferred to 0.1% gelatin coated dishes and grown in EGM-2 media for another 8 days. On day 13, greater than 12% of cells expressed CD31. (B) After 6 days of culture, CD31^+^ hESC-ECs expressed high levels of CD31, VE-cadherin, and vWF. Scale bar, 20 µm.

**Figure 2:**
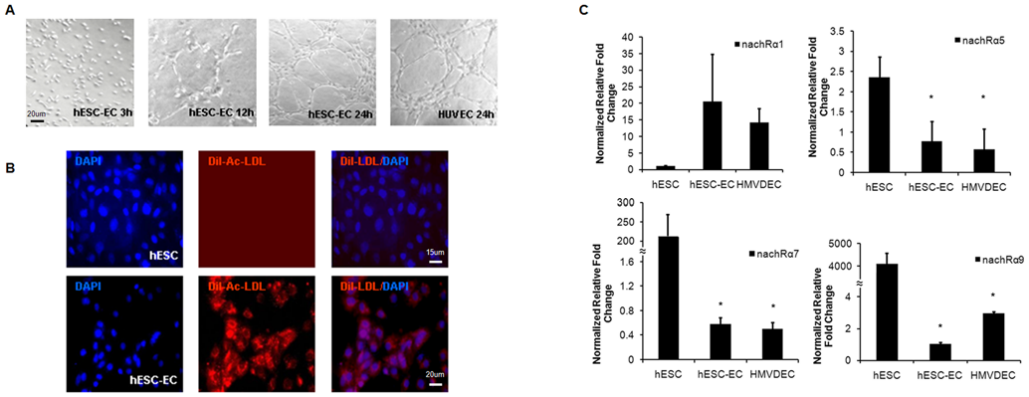
*In vitro* functional assessment and nAChR expression of hESC-ECs. (A) hESC-ECs formed visible tube-like structures beginning at 12 hours of culture on Matrigel. These structures were similar in morphology to human umbilical endothelial cells (HUVECs) used as positive control in this experiment (n = 8). (B) In contrast to undifferentiated hESCs, hESC-ECs were able to incorporate DiI-ac-LDL, a characteristic of mature endothelial cells. (C) Like human dermal microvascular endothelial cells (HMVDECs), hESC-ECs express similar levels of α1, α5, α7 and α9 nAChRs (n = 3). Scale bar, 20 µm.

To enhance the yield of endothelial cells from hESCs, a newly developed protocol was used to induce the differentiation of hESC-ECs by sequential treatment over 4 days with floating EB formation and 8 days of outgrowth differentiation from EBs in EGM-2 medium, which contains abundant growth factors to improve endothelial cell proliferation (**Figure 1A**). Compared with our previous hEB spontaneous differentiation method, which used 12-day floating hEB formation to induce differentiation (typically yielding <3% endothelial cells[15]), the floating/outgrowth hEB protocol consistently yielded >10% endothelial cells. After flow cytometry purification, cultures of highly pure vWF (96.3±1.4%), VE-cadherin (93.7±2.8%), and CD31 (97.2±2.5%) triple positive cell populations were readily obtained (**Figure 1B**). After further expansion in EGM-2 medium, these hESC-ECs were characterized for endothelial phenotype (i.e., ability to form tube-like structures in Matrigel and to incorporate DiI-ac-LDL), and for endothelial gene expression. After 12–24 hour incubation periods on Matrigel coated plates, we observed tube-like structures that were similar in morphology to human umbilical endothelial cells (HUVECs) (**Figure 2A**). In addition, DiI-ac-LDL uptake, which has been used to characterize endothelial cells [29], was avidly taken up by hESC-ECs (**Figure 2B**). In contrast, undifferentiated hESCs showed no uptake of DiI-ac-LDL. Quantitative real-time PCR expression of nAChRs in hESC-ECs and HMVDECs, expressed as relative fold changes with respect to hESCs, showed comparable gene expression levels for nAChR α1 (20.6±14.2 vs 14.2±0.1), α5 (0.8±0.2 vs 0.6±0.1), α7 (0.6±0.1 vs 0.5±0.1), and α9 (1.0±0.1 vs 3.0±2.2 (*P* = NS) (**Figure 2C**). Thus, these assays demonstrate that the purified hESC-ECs resemble mature endothelial cells and express nAChRs. Interestingly, when compared to hESCs, the hESC-ECs and HMVDECs showed significantly lower relative gene expression levels for nAChRs α5, α7 and α9 **(** **Figure 2C** **)**.

### Characterization of hESCs stably transduced with reporter genes for molecular imaging

**Figure 3:**
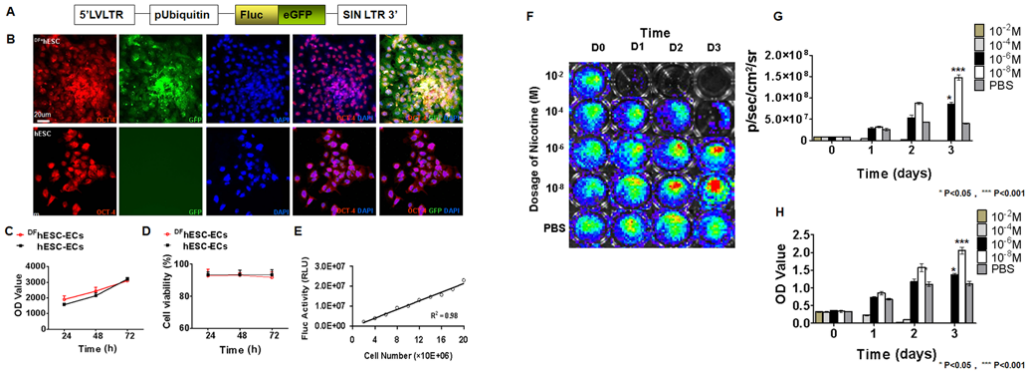
Stable lentiviral transduction of hESCs with double fusion (DF) reporter gene. (A) Schema of the DF reporter gene containing Fluc and eGFP. (B) Immunostaining of ^DF+^hESCs and control hESCs demonstrates similar expression of the stem cell marker Oct-4, while only ^DF+^hESCs express eGFP. ^DF+^hESC-ECs and non-transduced hESC-ECs had similar (C) cell proliferation and (D) cell viability rates over 72 hours, indicating that the Fluc reporter gene did not affect hESC characteristics. (E) A robust correlation exists between cell number and Fluc activity (R^2^ = 0.98). (F) 1×10^4^ hESC-ECs were cultured in the presence or absence of nicotine at 5 different concentrations (10^−2^, 10^−4^, 10^−6^, 10^−8^ M or control PBS over 3 days. (G) Quantification of BLI signal indicated that 10^−6^–10^−8^ M nicotine concentrations caused a significant enhancement in cell proliferation compared with other concentrations and PBS control group at day 3 (n = 8, *P<0.05, ***P<0.001). (H) Likewise, cell numbers were confirmed with serial spectrophotometer- based optical density (OD) which showed similar results. Scale bar, 20 µm.

In order to non-invasively track the effect of nicotine on hESC-EC survival and localization *in vivo*, hESCs were transduced with a double fusion (DF) reporter gene consisting of Fluc and eGFP (**Figure 3A**). Both the non-transduced hESCs and stably transduced ^DF+^hESCs showed similar expression patterns of stem cell markers Oct-4, whereas only transduced ^DF+^hESCs expressed eGFP as expected (**Figure 3B**). ^DF+^hESCs also showed similar proliferation and viability as non-transduced hESCs, suggesting no significant adverse effects by reporter gene expression (**Figure 3C–D**). We also observed a strong correlation (r^2^ = 0.98) between Fluc activity and ^DF+^hESC-EC numbers (**Figure 3E**). In order to assess the effect of nicotine on differentiated hESC-EC proliferation and reporter Fluc activity, 1×10^4^ control hESC-ECs and 1×10^4^ transduced hESC-ECs were cultured with or without the presence of various concentrations of nicotine (10^−2^, 10^−4^, 10^−6^, 10^−8^ M) for 24–72 hours (**Figure 3F–H**). BLI of Fluc activity as a measure of cell numbers suggested that pharmacological doses of nicotine (10^−2^–10^−4^ M nicotine) led to significant cell death within 24–72 hours, but interestingly clinically relevant concentrations of nicotine (10^−8^–10^−6^ M) enhanced cell survival at 72 hours (*P*<0.01). The enhancement of Fluc activity by clinically relevant concentrations of nicotine was further confirmed by MTS assay, suggesting that 10^−8^–10^−6^ M nicotine could enhance cell proliferation.

### nAChRs activation promotes survival of ^DF+^hESC-EC in ischemic myocardium

**Figure 4:**
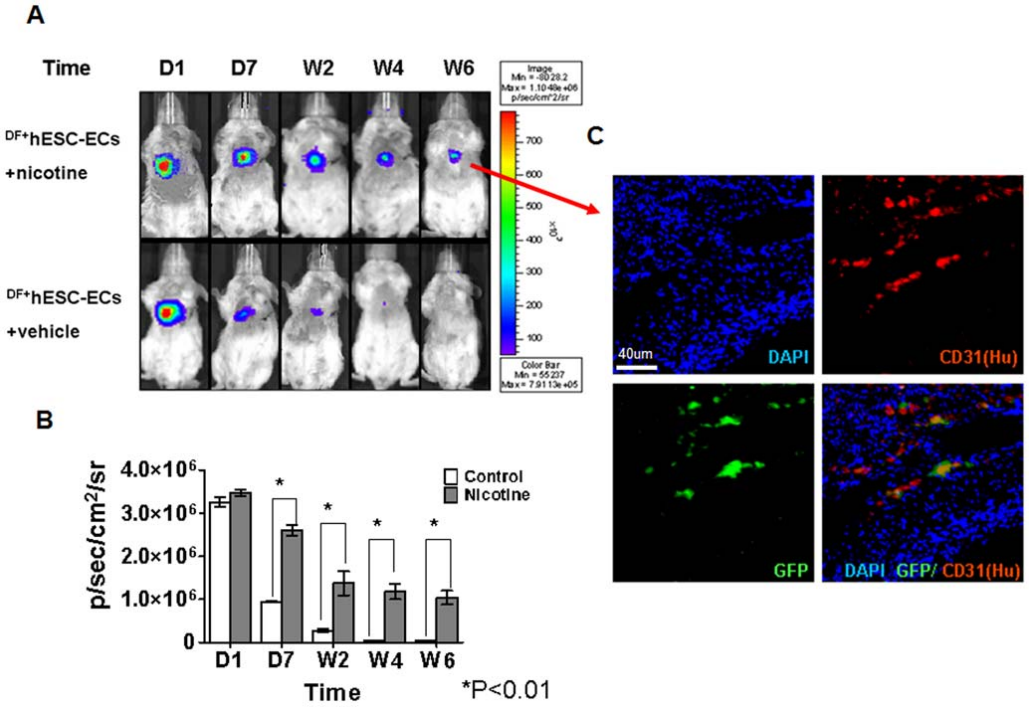
Activation of nAChRs promotes hESC-EC survival in ischemic hearts. (A) Following LAD ligation, ^DF+^hESC-ECs were injected into peri-infarct area in adult SCID mice. In the nicotine group, mice were given nicotine in their drinking water (100 ug/ml) for 2 weeks post-surgery. In the vehicle group, mice were maintained without nicotine administration. Serial BLI was used to evaluate the survival of transplanted cells on days 1, 7, weeks 2, 4 and 6. (B) Quantification of the resulting imaging signals indicated significant enhancement of cell survival in nicotine-treated group compared with vehicle group, starting from day 7 (*P<0.01). (C) Immunohistochemistry of myocardial tissue sections at week 6, in nicotine treated group, reveals double staining against human specific CD31 and GFP antibody, indicating that ^DF+^hESC-ECs took part in the formation of vessel-like structures.

**Figure 5:**
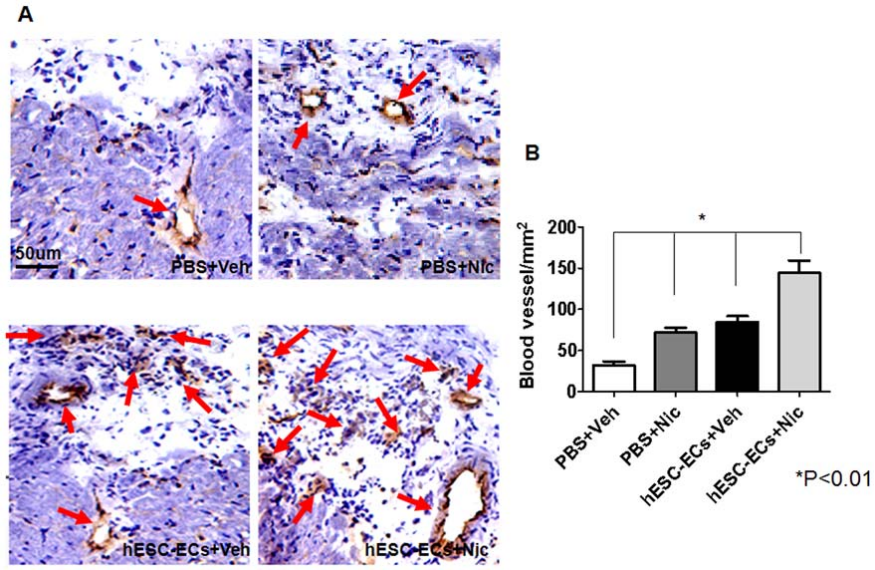
Effect of ^DF+^hESC-ECs transplantation and nicotine administration on neovascularization in the peri-infarct area. (A) Immunohistochemistry using mouse specific CD31 (brown color) was performed on tissue sections from animals receiving intramyocardial injections of ^DF+^hESC-EC or vehicle, in the presence or absence of nicotine treatment. (B) Quantification data showed ^DF+^hESC-EC transplantation plus 2 week nicotine administration group had the highest vessel density, compared with PBS + vehicle, PBS + nicotine, and ^DF+^hESC-EC + vehicle groups (*P<0.01) Scale bar, 50 µm.

To understand how nicotine might affect the survival and function of cardiac delivered hESC-ECs, animals received intramyocardial injections of 5×10^6^ ^DF+^hESC-ECs, and their diets supplemented with nicotine at a dose of 100 µg/ml in drinking water (**Figure 4A**). BLI was performed on days 1, 7, week 2, 4 and 6 after transplantation. Cell signal was most robust immediately after transplantation and gradually decreased both in the nicotine-treated (group 1) and vehicle groups (group 2) from day 2 to week 6 (**Figure 4B**). The short course of nicotine administration enhanced the long-term survival of implanted ^DF+^hESC-ECs. At week 6, immunohistochemistry showed vessel-like structures formed in part by ^DF+^hESC-ECs at the peri-infarct regions in the nicotine-treated group. These cells can be identified by double staining for CD31 (endothelial marker) and eGFP (reporter gene) as shown in (**Figure 4C**). Animals receiving intramyocardial injections of either 30 µl of 10^−8^ M nicotine (group 3) or 30 µl of PBS (group 4) but without hESC-ECs showed no BLI signals as expected (data not shown). In addition to enhancing cell survival, nAChR activation also promoted significantly higher levels of angiogenesis. Mouse myocardial neovascularization as assessed by microvessel density (MVD) was enhanced after receiving hESC-EC transplantation plus short-term nicotine treatment at 6 weeks after cell delivery (P<0.01), when compared to all other treatment groups (**Figure 5A–B**).

### Activation of nAChRs improve the angiogenic and anti-apoptotic potential of hESC-ECs through MAPK/Akt/HIF-1α pathway *in vitro*

**Figure 6:**
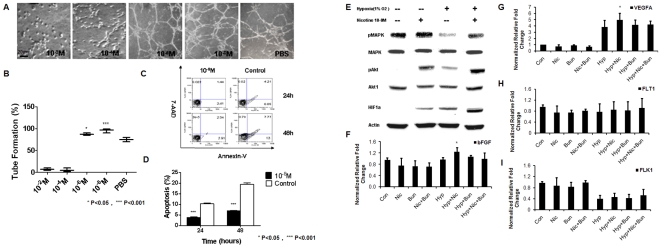
Nicotine's effects on hESC-EC apoptosis and angiogenesis in response to hypoxia *in vitro*. (A–B) hESC-ECs were seeded on GFR Matrigel-coated plate and incubated in a hypoxic condition (1% O_2_) with different concentrations of nicotine. 10^−8^ M nicotine resulted in significantly more tube-formation compared to other groups (n = 8, *P<0.05, ***P<0.001). (C–D) hESC-ECs were treated with 10^−8^ M nicotine or vehicle in hypoxic conditions. Flow cytometry analysis was then used to assess cell staining for 7-AAD (dead cell marker) and Annexin-V (apoptosis marker) after 24 and 48 hours. 10^−8^ M nicotine significantly attenuated the percentage of dead and apoptotic hESC-ECs at 24 and 48 hours (n = 8). (E) Western analysis revealed that hypoxia (for 48 hrs) reduced, and nicotine (10^−8^ M) restored, pMAPK expression. Hypoxia increased pAkt, as did nicotine to a greater extent. (F–I) Quantitative PCR revealed that nicotine further increased the expression of an upregulation of VEGF-A and bFGF, an effect that was abrogated by the nAChR antagonist bungarotoxin (Bun). Expression of FLT-1 and FLK-1 was unchanged by nicotine. Scale bar, 20 µm.

We next assessed whether nicotine imparted angiogenic or pro-survival cues to hESC-ECs in hypoxic environments. To test the effect of nicotine on the angiogenic potential of hESC-EC in hypoxia, we assayed for the formation of tube-like structures on growth factor reduced Matrigel in the setting of hypoxia (5% CO_2_, 1% O_2_, 94% N_2_ culture conditions). Under hypoxic conditions for 48 hours, we observed a significant reduction in tube-like structures when treated with pharmacological doses of nicotine (10^−2^ M or 10^−4^ M) (**Figure 6A**). In stark contrast, when we used clinically relevant doses of nicotine, we observed a significant increase in tube-like formation with a maximum response at 10^−8^ M nicotine (**Figure 6B**). To further elucidate the apparent positive effect of 10^−8^ M nicotine on hESC-EC survival, we next quantified the cellular apoptosis induced by hypoxia (1% O_2_). Using Annexin-V and 7-AAD staining to assess cell apoptosis and death, respectively, we found that 10^−8^ M nicotine significantly attenuated the percentage of apoptotic hESC-ECs compared with PBS: 4.7±1.4% vs. 11.3±2.1% after 24 hour hypoxia incubation and 7.2±1.0% vs. 19.5±3.8% after 48-hour hypoxia incubation (**Figures 6C–D**). However, proliferation was not affected by the presence of 10^−8^ M nicotine in hypoxic conditions (**Figure S1**). Furthermore, to preclude the possibility that nicotine and/or hypoxia may impart pro-survival cues by modulating nAChR gene expression, we showed by real-time PCR analysis that neither nicotine nor hypoxia significantly affected the gene expression of α1, α5, α7, and α9 nAChRs **(Figure S2)**. Taken together, these results show that the activation of nAChRs led to an anti-apoptotic effect and a pro-angiogenic effect of 10^−8^ M nicotine on hESC-ECs in hypoxic conditions.

To further investigate the mechanism governing the pro-survival effects of nicotine in hypoxia, we tested the effect of 10^−8^ M nicotine on the expression of MAPK, phosphorylated MAPK (pMAPK), Akt, phosphorylated Akt (pAkt), and hypoxia inducible factor-1 alpha (HIF-1α), all of which are involved in cell proliferation, survival, apoptosis, and angiogenesis (**Figure 6E** **, Figure S3**). The expression levels of both MAPK and Akt remained unchanged by nicotine in all treatment groups. However, after 48-hour exposure of the cells to hypoxia, 10^−8^ M nicotine enhanced the phosphorylation of MAPK. In both normoxic and hypoxic conditions, 10^−8^ M nicotine induced the phosphorylation of Akt, suggesting an effect of nAChR activation on these signaling molecules. HIF-1α was upregulated in hypoxia but showed non-significant trends of increased activation in the presence of nicotine. In addition to protein expression, quantitative PCR analysis revealed that nAChR activation during hypoxia could selectively up-regulate the angiogenic genes bFGF and VEGF-A, but not FLT1 or FLK1 expression **(** **Figure 6F–I** **, Table S1)**. This effect was blocked by the nAChR antagonist, α-bungarotoxin (α-BTX). These results suggest that nAChR activation in the presence of hypoxia could promote the up-regulation of VEGF and bFGF gene expression, and that MAPK and Akt phosphorylation is likely to be involved in nAChR-mediated effects on hESC-ECs.

To verify the role of Akt in modulating nicotine's enhancement in cell survival under hypoxic conditions, hESC-ECs were cultured in EBM in 1% O_2_ hypoxia in the presence or absence of 10^−8^ M nicotine for 24 h before being treated by 5 µM Akt IV inhibitor. As shown in **Figure S4**, the Akt IV inhibitor stimulated increased cell death when compared to the control group, and this effect was partially blunted by the co-administration of both Akt IV inhibitor and nicotine. This data suggests that inhibiting the Akt pathway led to increased cell death, and this effect could be partially reversed by nicotine. This finding concurs with our immunoblotting data by demonstrating the importance of Akt and nAChR activation for cell survival during hypoxia.

## Discussion

The ability to obtain a potentially unlimited source of endothelial cells from hESCs holds great promise for future regenerative medicine therapies. However, stem cell therapy for cardiovascular disease will ultimately be used in patients with risk factors such as tobacco smoking. We therefore were interested whether nicotine, a key component of tobacco smoke, would have any effect on the survival or incorporation of hESC-ECs into the ischemic heart. Surprisingly, short-term exposure to nicotine at clinically relevant concentrations (eg. in the nanomolar range) *enhanced* hESC-EC survival in the ischemic heart, and new vessel formation. Following intramyocardial injection of ^DF+^hESC-ECs, we observed a rapid decrease in imaging intensity over seven days in the control group, which indicated significant cell death. However, we were surprised to see significant prolongation of ^DF+^hESC-EC survival in the nicotine-treated group out to 5 weeks.

To understand the mechanism of nicotine-induced hESC-EC survival, we next performed a series of *in vitro* experiments using different concentrations of nicotine (10^−8^ M to 10^−2^ M). Significant cell death occurred at the higher concentrations (10^−4^–10^−2^ M), but at lower concentrations (10^−8^–10^−6^ M) we observed increased proliferation, anti-apoptosis, and angiogenesis. In particular, the 10^−8^ M concentration had the largest effect on proliferation. Our *in vitro* analysis suggests that as a non-selective agonist of nAChRs, nicotine (at the clinically relevant dose of 10^−8^ M) can improve hESC-EC angiogenesis and prevent apoptosis under hypoxia through MAPK and Akt signaling pathways. Furthermore, nAChR activation led to upregulation of VEGF-A and bFGF gene expression and enhanced hESC-EC survival and neovasculature formation after delivery in ischemic heart tissue. Interestingly, the finding that angiogenesis was upregulated after hESC-EC transplantation followed by low-dose nicotine administration suggests that the activation of nAChRs can improve the paracrine effect of hESC-EC on VEGF secretion, which is another benefit from stem cell therapy[30].

One limitation of this study is that ^DF+^hESC-ECs were used for *in vivo* studies, whereas non-transduced cells were assessed for cell survival and angiogenic effects. However, we have shown that the ^DF+^hESC-ECs cells exhibit similar proliferation, viability, and phenotypic markers as non-transduced cells (**Figure 3C–D** **,** **Figure 4C**). This result is in agreement with our previous reports that show that transduced hESCs maintain the pluripotent stem cell phenotype. Therefore, it is likely that both ^DF+^hESC-ECs and non-transduced hESC-ECs respond to nAChR activation in a similar manner.

Our investigation into nicotine's effects on stem cell survival is only the latest study of this drug after decades of research into its cellular and physiologic effects. In recent years, a growing body of evidence indicates that non-neuronal nAChRs, when activated by nicotine, may play a prominent role in endothelial cell survival, proliferation[31], and mobilization[32], through their angiogenic[25], [33], [34], anti-inflammatory[35], [36], and anti-apoptotic[37], [38] properties. Along with acetylcholine, nicotine is a ligand for nAChRs, which are cholinergic ion channels found in plasma membranes of many different cell types, primarily neurons. Evidence suggests that non-neuronal nAChRs are involved in the regulation of vital cell functions, such as mitosis, differentiation, organization of the cytoskeleton, cell-cell contact, locomotion, and migration [22], [25], [31], [34], [37]. Furthermore, at clinically relevant concentrations of nicotine (i.e., 1-100 nM range experienced by smokers or individuals treated with nicotine), nicotine has been shown to promote angiogenesis in a number of *in vivo* settings, including inflammation, wound healing, ischemia, tumor, and atherosclerosis[22], [25], [33], [34].

However, these unexpected effects of nicotine, along with their implications for cell-based therapies, are often countered by other compounds found in tobacco smoke. Though nicotine is a key addictive component of tobacco, it is notable that tobacco smoke consists of more than 4,000 chemicals[39], many of them carcinogenic or otherwise toxic. For example, the liquid vapor portion of the smoke aerosol contains the compounds acrolein[40] and benzopyrene[41], which are known to be cytotoxic and mutagenic, and may account for some of second-hand smoke's toxicities[42]. Nicotine is thus not the sole or even most important mediator of tobacco's harmful effects, and studies demonstrating its angiogenic and proliferative properties through nAChRs on endothelial cells have hinted at the intriguing and varied bio-activities of this compound.

In summary, this is the *first* study to investigate the effect of nAChRs on hESC-EC behavior both *in vitro* and *in vivo*, as well as the first study to elucidate the relationship between nAChRs activation and the downstream signaling pathways including MAPK, Akt, and HIF-1α. MAPK cascades are well known multi-functional signaling networks that influence cell growth, differentiation, apoptosis, and cellular responses to stress. HIF-1α is an important transcriptional factor that activates the gene expression of growth factors and promotes the expression of several genes which confer hypoxic tolerance through angiogenesis, erythropoeisis, vasodilation, and altered glucose metabolism. We have demonstrated *in vitro* that the activation of nAChRs by its ligand—a low dose of nicotine—can trigger anti-apoptotic, angiogenic, and proliferative pathway. Furthermore, systemic *in vivo* administrations of nicotine protected hESC-ECs from acute cell loss after transplantation to ischemic heart tissue. Taken together, we believe the activation of nAchRs has a potential positive role to play in regenerative medicine, and may become valuable for improving hESC-EC survival and, ultimately, therapeutic efficacy.

## Supporting Information

Figure S1 Effect of nicotine on cell proliferation in hypoxia *in vitro*. hESC-ECs were cultured in 1% O2 in the presence of PBS or 10^−8^ M nicotine for up to 2 days. (A) Cell proliferation was assayed by BrdU incorporation (green) and expressed as a percentage of total cell nuclei (blue). (B) Quantification of BrdU+ cells after 1 and 2 days. Data is shown as mean ± standard deviation (n = 3). Scale bar, 50 µm.
(3.18 MB TIF)

Figure S2 Effects of nicotine or hypoxia on the expression of nAChR subunits in hESC-ECs. Taqman real-time PCR showed no significant effect of 48-hour hypoxia or 10^−8^ M nicotine on α1, α5, α7 and α9 nAChR expression. Data is normalized to 18S housekeeping gene and expressed as fold changes ± standard deviation, relative to the normoxia treatment group (n = 3).
(3.18 MB TIF)

Figure S3 Effect of nicotine on the activation of signaling pathways. Quantification of immunoblots for (A) pMAPK,, (B) pAkt, and (C) HIF1α. Data for pMAPK and pAkt were normalized to total MAPK or Akt, respectively. HIF1α abundance was normalized to total actin. Data is shown as mean ± standard error of mean (n = 3).
(3.18 MB TIF)

Figure S4 Role of nicotine in Akt-mediated improvement of cell viability in hypoxia. After 24 h in the presence of hypoxia and nicotine, cells were incubated with 5 µM Akt IV inhibitor, 10^−8^ M nicotine, or Akt IV inhibitor + Nicotine for 1 h before assaying for cell viability (n = 4). Scale bar, 200 µm.
(3.18 MB TIF)

Table S1 Effect of nicotine on angiogenic genes expression. The downstream gene expression of both VEGF-A and bFGF were up-regulated, but FLT-1 and FLK-1 expression remained unchanged. Data is normalized to 18S housekeeping gene and expressed as fold changes ± standard deviation, relative to the normoxia treatment group (n = 4).
(3.18 MB TIF)

## References

[1] Thomson JA, Itskovitz-Eldor J, Shapiro SS, Waknitz MA, Swiergiel JJ (1998). Embryonic stem cell lines derived from human blastocysts.. Science.

[2] Shamblott MJ, Axelman J, Wang S, Bugg EM, Littlefield JW (1998). Derivation of pluripotent stem cells from cultured human primordial germ cells.. Proceedings of the National Academy of Sciences of the United States of America.

[3] Klimanskaya I, Rosenthal N, Lanza R (2008). Derive and conquer: sourcing and differentiating stem cells for therapeutic applications.. Nature Reviews Drug Discovery.

[4] Moon SY, Park YB, Kim D-S, Oh SK, Kim D-W (2006). Generation, culture, and differentiation of human embryonic stem cells for therapeutic applications.. Molecular Therapy.

[5] Lu S-J, Feng Q, Caballero S, Chen Y, Moore MAS (2007). Generation of functional hemangioblasts from human embryonic stem cells.. Nature Methods.

[6] Cho S-W, Moon S-H, Lee S-H, Kang S-W, Kim J (2007). Improvement of postnatal neovascularization by human embryonic stem cell derived endothelial-like cell transplantation in a mouse model of hindlimb ischemia.. Circulation.

[7] Wang ZZ, Au P, Chen T, Shao Y, Daheron LM (2007). Endothelial cells derived from human embryonic stem cells form durable blood vessels in vivo.. Nature Biotechnology.

[8] Ferreira LS, Gerecht S, Shieh HF, Watson N, Rupnick MA (2007). Vascular progenitor cells isolated from human embryonic stem cells give rise to endothelial and smooth muscle like cells and form vascular networks in vivo.. Circulation Research.

[9] Levenberg S, Zoldan J, Basevitch Y, Langer R (2007). Endothelial potential of human embryonic stem cells.. Blood.

[10] Laflamme MA, Chen KY, Naumova AV, Muskheli V, Fugate JA (2007). Cardiomyocytes derived from human embryonic stem cells in pro-survival factors enhance function of infarcted rat hearts.. Nature Biotechnology.

[11] Schulz TC, Palmarini GM, Noggle SA, Weiler DA, Mitalipova MM (2003). Directed neuronal differentiation of human embryonic stem cells.. BMC Neurosci.

[12] Jiang J, Au M, Lu K, Eshpeter A, Korbutt G (2007). Generation of insulin-producing islet-like clusters from human embryonic stem cells.. Stem Cells.

[13] Yamahara K, Sone M, Itoh H, Yamashita JK, Yurugi-Kobayashi T (2008). Augmentation of neovascularizaiton in hindlimb ischemia by combined transplantation of human embryonic stem cells-derived endothelial and mural cells.. PLOS One.

[14] Sone M, Itoh H, Yamahara K, Yamashita JK, Yurugi-Kobayashi T (2007). Pathway for differentiation of human embryonic stem cells to vascular cell components and their potential for vascular regeneration.. Arteriosclerosis, Thrombosis, and Vascular Biology.

[15] Li, Suzuki, Huang, Cao, Xie (2008). Comparison of reporter gene and iron particle labeling for tracking fate of human embryonic stem cells and differentiated endothelial cells in living subjects.. Stem Cells.

[16] Cao F, Lin S, Xie X, Ray P, Patel M (2006). In vivo visualization of embryonic stem cell survival, proliferation, and migration after cardiac delivery.. Circulation.

[17] Swijnenburg R-J, Schrepfer S, Govaert JA, Cao F, Ransohoff K (2008). Immunosuppressive therapy mitigates immunological rejection of human embryonic stem cell xenografts.. Proceedings of the National Academy of Sciences of the United States of America.

[18] Heiss C, Amabile N, Lee AC, Real WM, Schick SF (2008). Brief secondhand smoke exposure depresses endothelial progenitor cells activity and endothelial function: sustained vascular injury and blunted nitric oxide production.. Journal of the American College of Cardiology.

[19] Chen T, Bai H, Shao Y, Arzigian M, Janzen V (2007). Stromal cell-derived factor-1/CXCR4 signaling modifies the capillary-like organization of human embryonic stem cell-derived endothelium in vitro.. Stem Cells.

[20] Sugimoto A, Masuda H, Eguchi M, Iwaguro H, Tanabe T (2007). Nicotine enlivenment of blood flow recovery following endothelial progenitor cell transplantation into ischemic hindlimb.. Stem Cells and Development.

[21] Li Z, Wu JC, Sheikh AY, Kraft D, Cao F (2007). Differentiation, survival, and function of embryonic stem cell derived endothelial cells for ischemic heart disease.. Circulation.

[22] Heeschen C, Chang E, Aicher A, Cooke JP (2006). Endothelial progenitor cells participate in nicotine-mediated angiogenesis.. Journal of the American College of Cardiology.

[23] Levenberg S, Huang NF, Lavik E, Rogers AB, Itskovitz-Eldor J (2003). Differentiation of human embryonic stem cells on three-dimensional polymer scaffolds.. Proceedings of the National Academy of Sciences of the United States of America.

[24] Michaud S-E, Menard C, Guy L-G, Gennaro G, Rivard A (2003). Inhibition of hypoxia-induced angiogenesis by cigarette smoke exposure: impairment of the HIF-1alpha/VEGF pathway.. The FASEB Journal.

[25] Heeschen C, Weis M, Aicher A, Dimmeler S, Cooke JP (2002). A novel angiogenic pathway mediated by non-neuronal nicotinic acetylcholine receptors.. The Journal of Clinical Investigation.

[26] Xie X, Cao F, Sheikh AY, Li Z, Connolly AJ (2007). Genetic modification of embryonic stem cells with VEGF enhances cell survival and improves cardiac function.. Cloning and Stem Cells.

[27] Shetzline SE, Rallapalli R, Dowd KJ, Zou S, Nakata Y (2004). Neuromedin U: a Myb-regulated autocrine growth factor for human myeloid leukemias.. Blood.

[28] Livak KJ, Schmittgen TD (2001). Analysis of relative gene expression data using real-time quantitative PCR and the 2(-Delta Delta C(T)) Method.. Methods.

[29] Voyta JC, Via DP, Butterfield CE, Zetter BR (1984). Identification and isolation of endothelial cells based on their increased uptake of acetylated-low density lipoprotein.. J Cell Biol.

[30] Gnecchi M, Zhang Z, Ni A, Dzau VJ (2008). Paracrine mechanisms in adult stem cell signaling and therapy.. Circulation Research.

[31] Villablanca AC (1998). Nicotine stimulates DNA synthesis and proliferation in vascular endothelial cells in vitro.. Journal of Applied Physiology.

[32] Chen C, Ridzon D, Lee C-T, Blake J, Sun Y (2007). Defining embryonic stem cell identity using differentiation-related microRNAs and their potential targets.. Mammalian Genome.

[33] Cooke JP (2007). Angiogenesis and the role of the endothelial nicotinic acetylcholine receptor.. Life Sciences.

[34] Heeschen C, Weis M, Cooke JP (2003). Nicotine promotes arteriogenesis.. Journal of the American College of Cardiology.

[35] Sadis C, Teske G, Stokman G, Kubjak C, Claessen N (2007). Nicotine protects kidney from renal ischemia/reperfusion injury through the cholinergic anti-inflammatory pathway.. PLOS One.

[36] Mills CM, Hill SA, Marks R (1997). Transdermal nicotine suppresses cutaneous inflammation.. Archives of dermatology.

[37] Dasgupta P, Kinkade R, Joshi B, Decook C, Haura E (2006). Nicotine inhibits apoptosis induced by chemotherapeutic drugs by up-regulating XIAP and survivin.. Proceedings of the National Academy of Sciences of the United States of America.

[38] Suzuki J, Bayna E, Dalle Molle E, Lew WYW (2003). Nicotine inhibits cardiac apoptosis induced by lipopolysaccharide in rats.. Journal of the American College of Cardiology.

[39] Burns DM (1991). Cigarettes and cigarette smoking.. Clinics in Chest Medicine.

[40] Finkelstein EI, Nardini M, van der Vliet A (2001). Inhibition of neutrophil apoptosis by acrolein: a mechanism of tobacco-related lung disease?. American journal of physiology Lung Cellular and Molecular Physiology.

[41] Rodriguez JW, Kirlin WG, Wirsiy YG, Matheravidathu S, Hodge TW (1999). Maternal exposure to benzo[a]pyrene alters development of T lymphocytes in offspring.. Immunopharmacology and Immunotoxicology.

[42] Zhu B-q, Heeschen C, Sievers RE, Karliner JS, Parmley WW (2003). Second hand smoke stimulates tumor angiogenesis and growth.. Cancer Cell.

