# Supplementary figures and images for: nAChRs Mediate Human Embryonic Stem Cell-Derived Endothelial Cells: Proliferation, Apoptosis, and Angiogenesis
Source: PLoS One. 2009 Sep 15;4(9):e7040. doi: 10.1371/journal.pone.0007040 (PMC2737633; doi:10.1371/journal.pone.0007040)

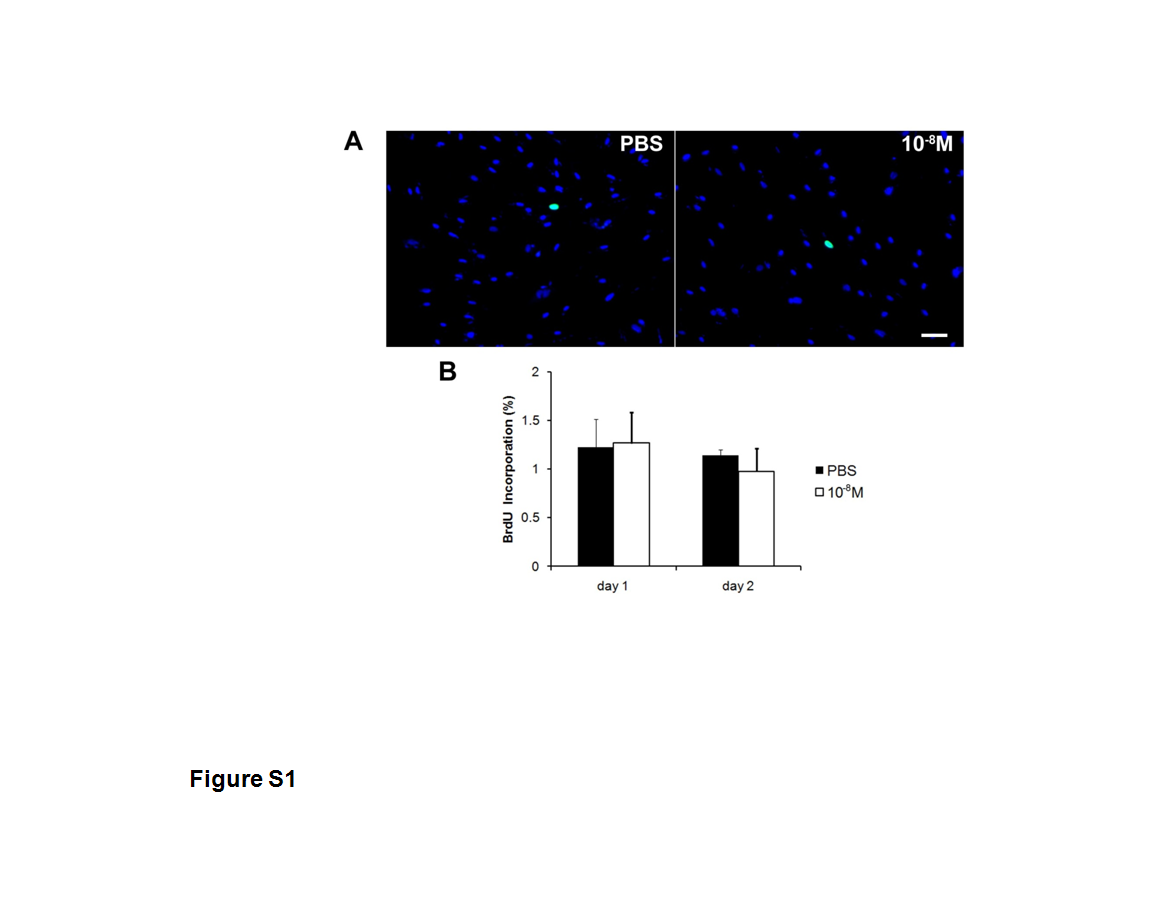

Supplement: Figure S1 — Effect of nicotine on cell proliferation in hypoxia in vitro. hESC-ECs were cultured in 1% O2 in the presence of PBS or 10−8 M nicotine for up to 2 days. (A) Cell proliferation was assayed by BrdU incorporation (green) and expressed as a percentage of total cell nuclei (blue). (B) Quantification of BrdU+ cells after 1 and 2 days. Data is shown as mean ± standard deviation (n = 3). Scale bar, 50 µm. (3.18 MB TIF) [file pone.0007040.s001.tif]

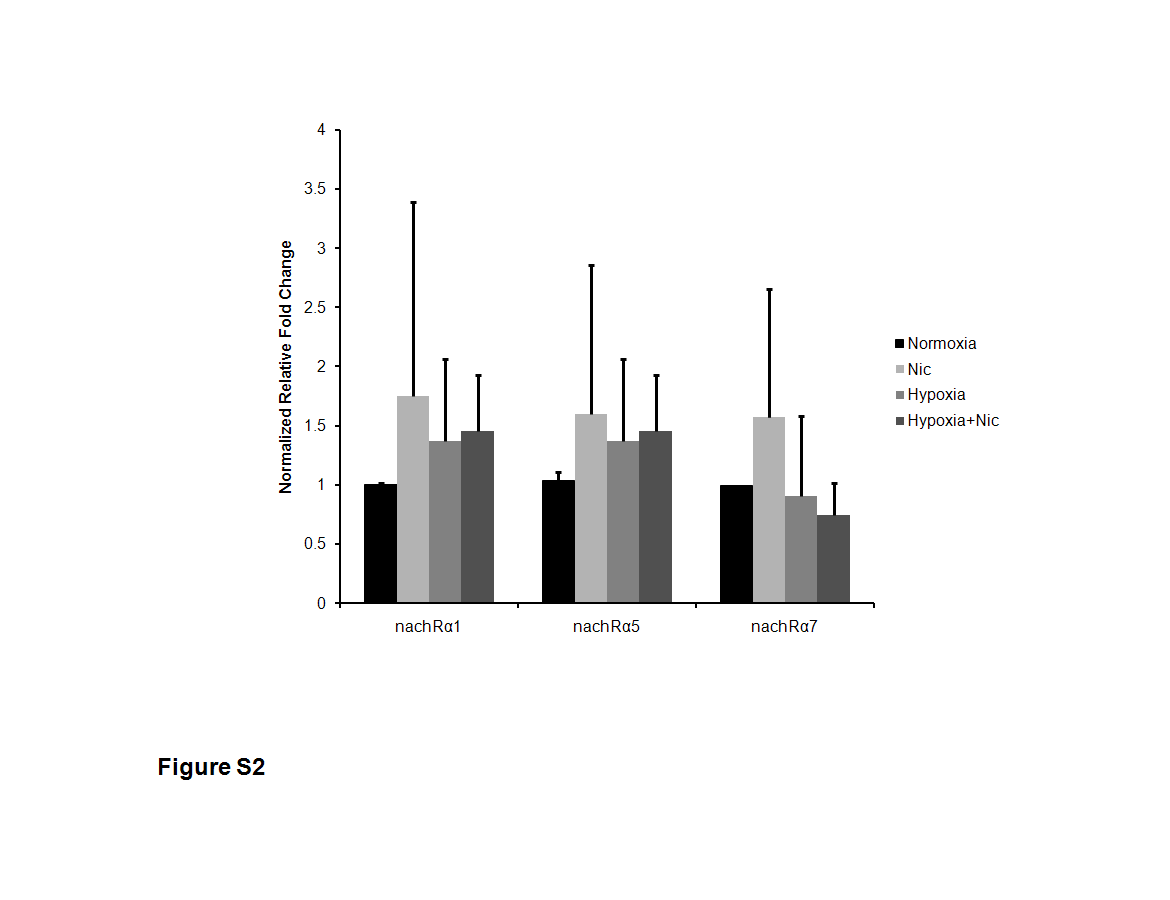

Supplement: Figure S2 — Effects of nicotine or hypoxia on the expression of nAChR subunits in hESC-ECs. Taqman real-time PCR showed no significant effect of 48-hour hypoxia or 10−8 M nicotine on α1, α5, α7 and α9 nAChR expression. Data is normalized to 18S housekeeping gene and expressed as fold changes ± standard deviation, relative to the normoxia treatment group (n = 3). (3.18 MB TIF) [file pone.0007040.s002.tif]

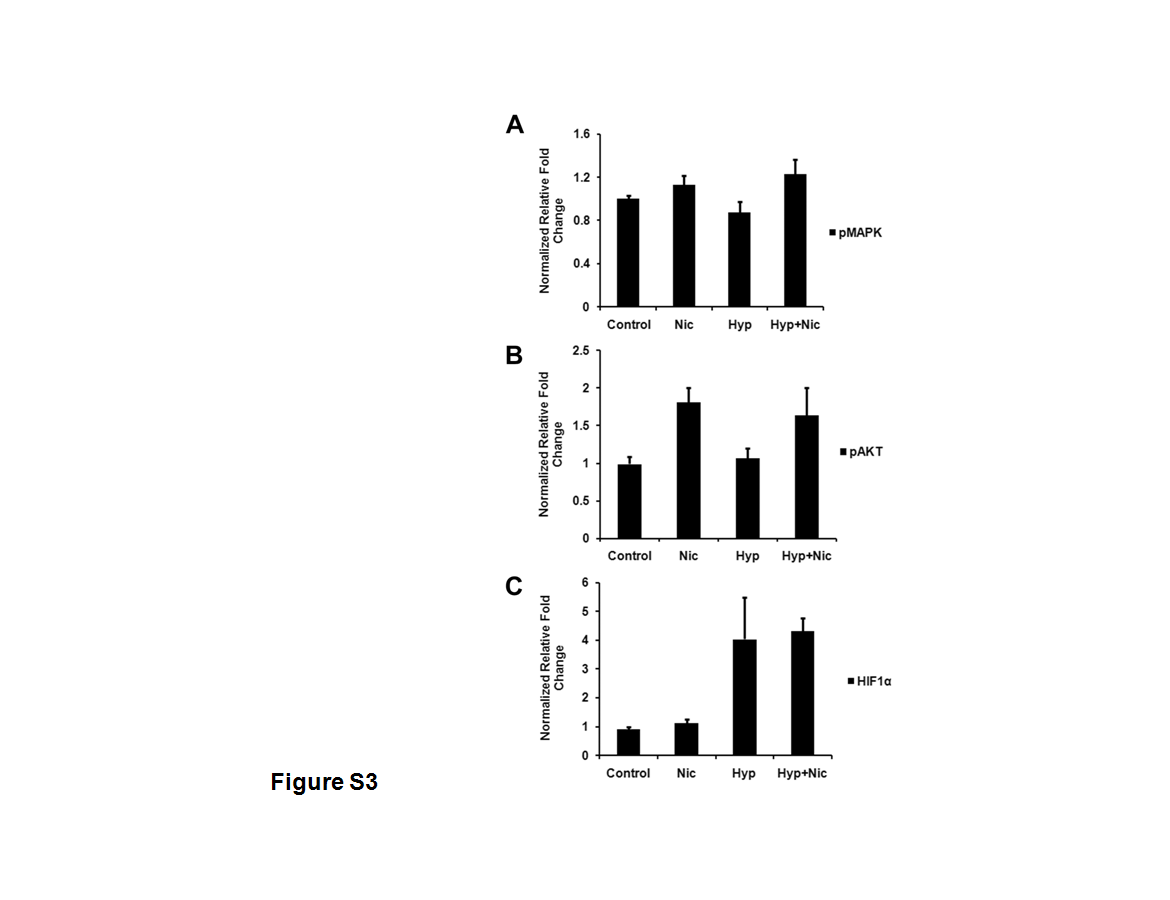

Supplement: Figure S3 — Effect of nicotine on the activation of signaling pathways. Quantification of immunoblots for (A) pMAPK,, (B) pAkt, and (C) HIF1α. Data for pMAPK and pAkt were normalized to total MAPK or Akt, respectively. HIF1α abundance was normalized to total actin. Data is shown as mean ± standard error of mean (n = 3). (3.18 MB TIF) [file pone.0007040.s003.tif]

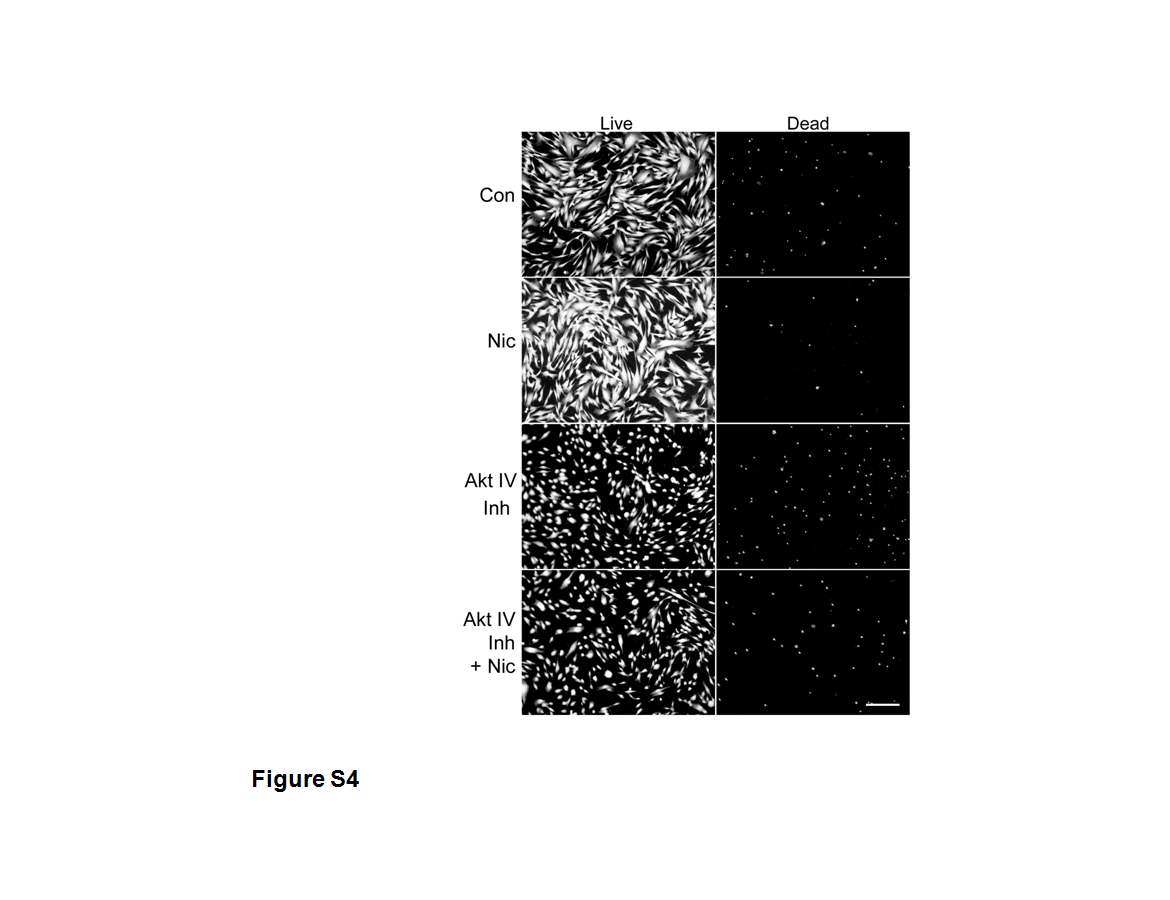

Supplement: Figure S4 — Role of nicotine in Akt-mediated improvement of cell viability in hypoxia. After 24 h in the presence of hypoxia and nicotine, cells were incubated with 5 µM Akt IV inhibitor, 10−8 M nicotine, or Akt IV inhibitor + Nicotine for 1 h before assaying for cell viability (n = 4). Scale bar, 200 µm. (3.18 MB TIF) [file pone.0007040.s004.tif]

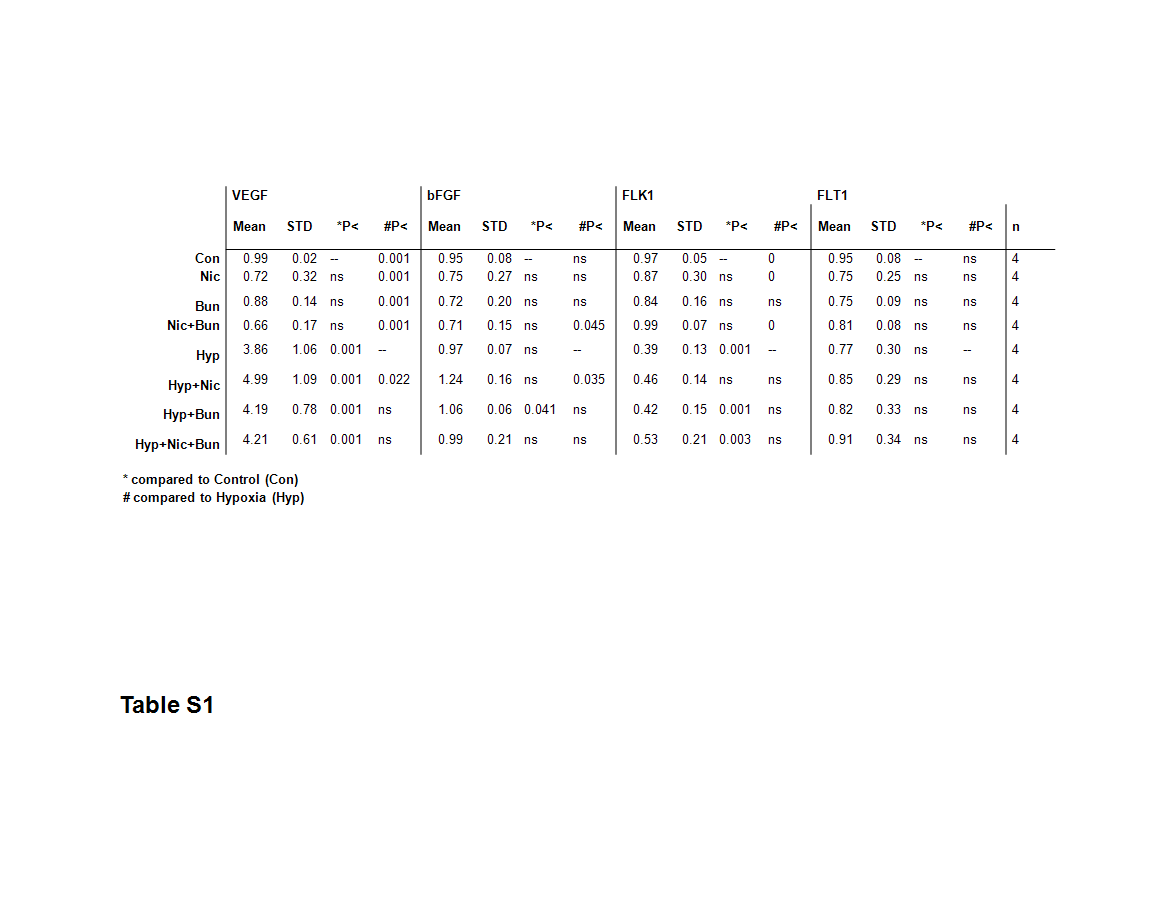

Supplement: Table S1 — Effect of nicotine on angiogenic genes expression. The downstream gene expression of both VEGF-A and bFGF were up-regulated, but FLT-1 and FLK-1 expression remained unchanged. Data is normalized to 18S housekeeping gene and expressed as fold changes ± standard deviation, relative to the normoxia treatment group (n = 4). (3.18 MB TIF) [file pone.0007040.s005.tif]
